# Case of Rupture of the Uterus, with Occlusion of the Vagina

**Published:** 1844-05

**Authors:** Daniel Brainard


*Case of Rupture of the Uterus, with occlusion of the Vagina.* By DANIEL BRAINARD, M. D.

*On Monday, April* 8, 1844, I was invited by Dr. J. Brinckerhoff, a highly respectable practitioner of this city, to examine the body of Mrs. Donnahue, who had died during labor. The history of the case was as follows:

*On Tuesday, April* 2d, at the full period of gestation, she was taken in labor, and the Dr. was called, who finding but slight pains, and there being constipation of the bowels, administered a cathartic of castor oil, after the operation of which, the pains entirely subsided, and on Thursday, she felt quite well and went about as formerly. Friday, at 2 A. M., pains returned, were of ordinary severity, and irregular in their returns, waters discharged, a venesection was practiced, and the physician was absent at times during the day. Saturday, at 4 o'clock, A. M., he was called in haste, but on his arrival, found the pains had suddenly left her, and that she complained of fullness at the epigastric region. Tenderness of the abdomen supervened, with frequency of the pulse and great depression, and death took place on Monday, at 2 A. M., The examination was made at 10 o'clock of the same morning, and gave the following results:

The vagina was closed above the middle by adhesions, which seemed perfectly to have obstructed the passage, and a firm band was found to extend from the left side of it, upward and backward, to its termination. No trace of the os uteri or of the head of the child could be felt. The abdomen having been laid open, the child, which was of full size, was brought into view. presenting its back to the anterior wall, the vertex resting in the left iliac fossa, the chin above the symphysis pubis, and the breech at the ensiform cartilage. The right iliac fossa was occupied by the placenta and membranes. The peritoneum was, at several points, of a deep red color, coated with a thin layer of coagulable lymph, and contained a small quantity of lymph and serum. On removing the child, the uterus was seen, of its usual size after delivery, slightly depressed into the pelvis. Drawing it gently upward, a rent was perceived at its anterior part, immediately above the attachment of the vagina, extending from side to side. Passing the finger through this into the vagina, it was arrested, as below, by the adhesions, and with a finger on either side, the septum appeared to be half an inch in thickness, and of very firm texture. The uterus and vagina, having been removed for more careful examination, the margins of the rent were found to be rough and ragged, numerous large vessels were seen upon the torn surfaces, which were quite empty, and many fibres were attached to them drawn out from the opposite sides, which, to the unassisted eye, appeared like those of a muscle which has been for some time macerated in water. The wall at the side of the opening was an inch in thickness, and presented no trace of any former disease. The cavity of the uterus contained a small coagulum. The closure of the vagina was found to be perfect, with the exception of a canal through which a quill of small size might be forced, the orifices of which were obscure. The septum itself was very dense, and composed of the fibrous tissue of cicatrix.

In regard to the previous history of this individual, but little accurate information could be obtained. She was 28 years of age, robust, had borne 2 children, the first of which was still born at full term, and after a severe labor, the second also still born, but after a premature labor at 7 months; at this second time she was in labor only four hours, and suffered but little. Such were the only facts I was able to ascertain, and they by no means account for the adhesions of the vagina.

The rule of conduct to be adopted in cases similar to the present, is an interesting subject for consideration. The division of the adhesions, when they come to be pressed upon by the child's head, is the course which would most generally be preferable. There might, however, be a case in which the time of performing this would be difficult to select, where, as in the present instance, there was doubt in the mind of the practioner if the full term of gestation was passed, which would induce him to defer it. Even if performed, the operation might not save the life of the patient. A case similar to this is recorded by Dr. Lombard, of Geneva, in which death from rupture of the uterus took place, although an attempt was made to separate the adhesions.

The removal of the child by the Cesarian section would have been admissable immediately after the rupture, if the physician had been present and recognized the accident at the time of its occurrence; but the small size of the canal of the vagina at the point of its contraction, would, by preventing the discharge of coagula and of the lochia, have favored the passage of these into the peritoneal cavity, and thereby have rendered the operation most hazardous. It is probable, therefore, that in this instance the advice of Hunter, Denman and others, (which they extended to all cases of rupture of the uterus,) viz: to leave the case to nature, deplorable as was the result, was the best that could have been adopted.

